# Comparative Efficacy of Chimeric Porcine Circovirus (PCV) Vaccines against Experimental Heterologous PCV2d Challenges

**DOI:** 10.3390/vetsci10020080

**Published:** 2023-01-21

**Authors:** Pichanun Wongchanapai, Panuwat Yamsakul, Jirapat Arunorat, Thunyamas Guntawang, Tidaratt Sittisak, Saralee Srivorakul, Kornravee Photichai, Roongroje Thanawongnuwech, Manakorn Sukmak, Kidsadagon Pringproa

**Affiliations:** 1 Department of Veterinary Biosciences and Veterinary Public Health, Faculty of Veterinary Medicine, Chiang Mai University, Chiang Mai 50100, Thailand; 2 Swine Business Unit, Zoetis (Thailand) Limited, Bangkok 10500, Thailand; 3 Department of Food Animal Clinic, Faculty of Veterinary Medicine, Chiang Mai University, Chiang Mai 50100, Thailand; 4 Center of Veterinary Diagnosis and Technology Transfer, Faculty of Veterinary Medicine, Chiang Mai University, Chiang Mai 50100, Thailand; 5 Department of Pathology, Faculty of Veterinary Sciences, Chulalongkorn University, Bangkok 10330, Thailand; 6 Department of Farm Resources and Production Medicine, Faculty of Veterinary Medicine, Kasetsart University, Nakorn Pathom 73140, Thailand; 7 Center of Excellence in Elephant and Wildlife Research, Chiang Mai University, Chiang Mai 50100, Thailand

**Keywords:** Porcine circovirus type 2, challenge, chimeric vaccine, heterologous PCV2

## Abstract

**Simple Summary:**

Disease caused by infection with porcine circovirus type 2 (PCV2), collectively known as porcine circovirus-associated disease (PCVAD), is one of the most important viral infectious diseases in pigs. To date, PCV2 has been classified into at least 8 genotypes, namely PCV2a, PCV2b, PCV2c, PCV2d, PCV2e, PCV2f, PCV2g, and PCV2h. Among these, PCV2a, PCV2b, and PCV2d are the predominant genotypes that have chronologically circulated and affected the global pig population. Application of the PCV2 vaccine is a key strategy in the prevention and control of PCV2 infection. However, to the best of our knowledge, little is known about the benefits of using the chimeric PCV2a-2b antigen-based vaccine in Thailand in experimental challenges with field isolates of PCV2d. The present study has demonstrated that the chimeric PCV1-2a-based vaccine and the chimeric PCV1-2a-2b-based vaccine are effective against Thai PCV2d inoculation. The present study further strengthens the use of the PCV2 vaccine as an important tool for prevention of PCVAD in pigs.

**Abstract:**

The objective of this study was to evaluate the efficacy of two multivalent commercial porcine circovirus (PCV) vaccines against heterologous PCV2d challenges. A total of 24 crossbred male pigs aged 26 days selected from a specific pathogen-free herd were randomly divided into four groups (six pigs per group) and assigned as follows: negative control (unvaccinated/sham-challenge), vaccinated with chimeric PCV1-2a vaccine (PCV1-2a/PCV2d-challenge), vaccinated with chimeric PCV1-2a-2b vaccine (PCV1-2a-2b/PCV2d-challenge) and positive control (unvaccinated/PCV2d-challenge). At 21 days after vaccination, the pigs were intranasally and intramuscularly inoculated with either sham or field isolates of PCV2d (PCV2d/149/TH/2020). After being challenged, blood samples were obtained weekly and analyzed for levels of PCV2d viremia, neutralizing antibodies, and IgG against PCV2. At 30 days post-challenge (DPC), the pigs were euthanized and then subjected to pathological evaluations and molecular analysis. The results indicated that pigs in the PCV1-2a-2b/PCV2d-challenge and the PCV1-2a/PCV2d-challenge groups possessed significantly greater levels of PCV2d-neutralizing antibody titer when compared with the positive control group. Moreover, pigs in the PCV1-2a-2b/PCV2d-challenge group exhibited a lower degree of severity in terms of gross lesion scores and lower levels of PCV2 viremia when compared with the positive control group. This study demonstrated that vaccinating pigs with either the PCV1-2a or PCV1-2a-2b chimeric vaccines elicits a potent immune response against PCV2d infection and reduces viremia after PCV2d inoculation in pigs.

## 1. Introduction

Porcine circovirus type 2 (PCV2), a single-strand non-enveloped DNA virus belonging to the family *Circoviridae*, genus *Circovirus*, has negatively impacted the pig industry worldwide [1,2]. PCV2 is known to be the primary causative agent of several syndromes in pigs that are collectively known as porcine circovirus-associated disease (PCVAD) [1]. To date, PCV2 has been classified into at least 8 genotypes, namely PCV2a, PCV2b, PCV2c, PCV2d, PCV2e, PCV2f, PCV2g, and PCV2h [3]. Among these, PCV2a, PCV2b, and PCV2d are the predominant genotypes that have chronologically circulated and affected the global pig population [3,4,5,6]. In Thailand, the genetic diversity of PCV2 has been reported on 56 farms, as has been confirmed by collection of 306 samples between 2009 and 2015 [7]. The results indicated that the prevalence of PCV2a was 5.5% and that of PCV2b was found to be 29.41%, while the prevalence of intermediate clade 1 (IM1) PCV2b was determined to be 11.03% and that of PCV2d was found to be 54.41% [7].

Application of the PCV2 vaccine is a key strategy in the prevention and control of PCV2 infection, as it has been determined that clinical signs of PCVAD were reduced under both experimental and field conditions [8,9,10,11,12]. Moreover, finishing pigs vaccinated with PCV2 vaccines experienced a significant increase in average daily gain (ADG) of 41.5 g/day and a significant decrease in mortality rate of 4.4% when compared with pigs of the unvaccinated herds [13]. It has also been demonstrated that coinfection of PCV2 with other pathogens in pigs has commonly occurred, leading to severe clinical signs of PCVAD under field conditions [1,2]. Thus, recent PCV2 vaccine research has been focused on the use of PCV2 antigen combination with other pathogens such a porcine parvovirus and *Mycoplasma hyopneumoniae* or with other PCV2 genotypes [14]. Interestingly, previous results indicate that PCV2a-2b antigen-based vaccines still have the efficacy to increase immunological responses and reduce the presence of PCV2 viremia after challenge [14,15]. This finding corresponds to those of a previous in silico study that used T-cell epitope content comparison (EpiCC) analysis to predict the similarity of the antigenic epitopes on the surface of the vaccine antigen and the antigenic epitopes on the pathogens obtained from field isolates [16]. The results of the EpiCC analysis indicate that cross protection may have occurred between the vaccine and the field virus. The outcomes of the study have also demonstrated that a combination of the PCV2a and PCV2b antigen-based vaccines had a higher EpiCC score when compared with the PCV2a antigen alone [16]. However, to the best of our knowledge, little is known about the benefits of using the PCV2a-2b antigen-based vaccine in Thailand in experimental challenges with field isolates of PCV2d. In the present study, we aimed to evaluate the use of the PCV2a-2b antigen-based vaccine by inoculation of the PCV2d virus collected from sick pigs living within Thai herds after administration of the PCV2a-2b antigen-based vaccine. 

## 2. Materials and Methods

### 2.1. Animals and Experimental Designs

The G power software (ver. 3.1.9.7; Heinrich-Heine-Universität Düsseldorf, Düsseldorf, Germany; http://www.gpower.hhu.de/ (accessed on 1 December 2020)) was used to determine the sample size in this study. Twenty-four crossbred male pigs aged 26 days obtained from a PRRSV-negative herd were raised in the Animal Biosafety Level (ABSL)-2 Room at the Laboratory Animal Center, Chiang Mai University, Thailand. Upon arrival, they were blood sampled and tested for the absence of PCV2 using a PCR test and tested for the absence of PRRSV using an enzyme-linked immunosorbent assay (ELISA) in conjunction with a PCR test. Pigs were then randomly divided into 4 groups, with 6 pigs/group. Animal protocols were conducted according to the Institute of Animal Care and Use Committee (approval number BR001/2565(03/2564-11-29), Chiang Mai University, Thailand. The weight of each pig was measured on the day of arrival and then every week. Final weights were measured when the pigs reached 75 days of age (28 days after PCV2d inoculation). The average daily gain (ADG) of each group was then analyzed. The experimental design and vaccination plan are presented in Table 1.

### 2.2. PCV2d Challenge

At 21 days post-vaccination, all pigs, with the exception of the unvaccinated/sham-challenged pigs, were inoculated with 10^5^ 50% tissue culture infectious dose (TCID_50_) per mL of a low-virulence PCV2d (PCV2d/149/TH/2020). To mimic the field situation, virus inoculation was accomplished with 2 mL intranasal and 2 mL intramuscular administrations of PCV2d in each pig, as has been previously described [17]. Blood samples were obtained weekly and analyzed for relevant antibody responses, as will be described below. At 28 days post-challenge (DPC), the final weights of all pigs were determined. At 30 DPC (77 days old), all pigs were euthanized and complete necropsy was then performed (Supplementary Materials). 

### 2.3. Immunological Response Evaluation and PCV2 DNA Detection

Sera were collected from all pigs weekly throughout the entire course of the study to evaluate the PCV2d-neutralizing antibodies using the immunoperoxidase monolayer assay (IPMA), as has been previously described [18]. In addition, PCV2-specific immunoglobulin G antibody (IgG) titers were also evaluated pre- and post-vaccination, and after being challenged, with indirect ELISA (BioCheck, ME, USA), as was outlined in the manufacturer’s instructions. Subsequently, the number of PCV2-DNA positive animals was determined by real-time PCR, as has been previously described [19]. 

### 2.4. Histopathology and Immunohistochemistry

The lung tissues, superficial inguinal lymph nodes, tracheobronchial lymph nodes, and mesenteric lymph nodes of the pigs were examined, collected, and subjected to immunohistochemistry to determine the histopathology, as has been previously described [20]. Briefly, tissue samples were deparaffined with xylene and rehydrated through serial graded ethanol. Endogenous peroxidase activity was then blocked by incubation with 3% hydrogen peroxide in distilled water. Tissue sections were then rinsed in 0.1 M Tris-buffered saline and incubated with 20% normal goat serum solution in 0.1 M Tris-buffered saline for 30 min at room temperature. Monoclonal anti-PCV2 antibody was used at a dilution of 1:200 in 0.1 M Tris-buffered saline and incubated. Biotinylated goat anti-mouse antibody was used as a secondary antibody and incubated for 1 h at room temperature. Then, avidin–biotin peroxidase (ABC; Thermo Scientific^®^, Waltham, MA, USA) reagent was applied for one hour at room temperature. Sections were finally incubated in diaminobenzidine (DAB)–hydrogen peroxide solution. The tissue samples were then counterstained with hematoxylin and dehydrated. Lastly, the slides were covered and examined microscopically. 

### 2.5. Immunohistochemistry Scoring

Immunohistochemical scoring of the PCV2 immunolabelling-positive cells was done by three pathologists, as has been described previously [21]. Briefly, tissue samples of the lungs, superficial inguinal lymph nodes, tracheobronchial lymph nodes, and mesenteric lymph nodes were classified into 4 categories, for which grade 0 was indicative of no signal, grade 1 indicated 0–25% of the positive signal in the studied areas, grade 2 indicated 25–50% of the positive signal in the studied areas, and grade 3 was indicative of having more than 50% of the positive signal in the studied areas. Average scores of the immunolabelling-positive cells were presented as mean ± standard error (SE). 

### 2.6. Statistical Analyses

Pig growth performance and immunohistochemistry scoring were analyzed by one-way analysis of variance (ANOVA). Results of the PCV2d-specific neutralizing antibody titers and the PCV2-specific IgG titers were analyzed by linear regression. The statistical analyses were accomplished using GraphPad Prism 5 (GraphPad Inc., La Jolla, CA, USA). Statistical significance was designated as *p* ≤ 0.05.

## 3. Results 

### 3.1. Growth Performance and Pathological Evaluation

**Figure 1:**
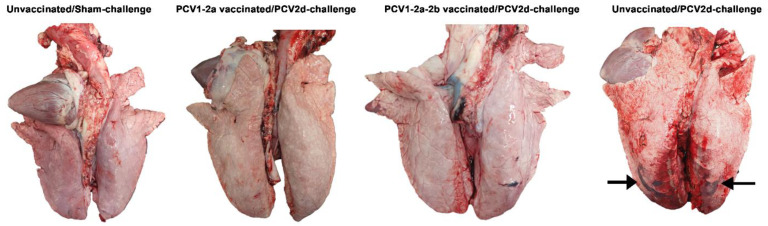
Macroscopically, predominant lesions of hemorrhagic pneumonia were observed in the unvaccinated/PCV2d-challenge pigs (arrows), while pigs in the PCV1-2a/PCV2d-challenge and PCV1-2a-2b/PCV2d-challenge groups displayed symptoms of mild hemorrhagic pneumonia.

In the present study, overt clinical signs of PCVAD were not observed among the pigs in the studied groups. The average daily weight gains (ADG) of the PCV1-2a and PCV1-2a-2b vaccinated pigs tended to be higher than those of the unvaccinated control group but were not statistically significant (Table S1). Macroscopically, the lungs of the PCV1-2a/PCV2d-challenge and the PCV1-2a-2b/PCV2d-challenge pigs exhibited no remarkable lesions, while pigs in the unvaccinated/PCV2d-challenge group were diagnosed with mild to moderate hemorrhagic broncho-interstitial pneumonia (Figure 1). 

**Figure 2:**
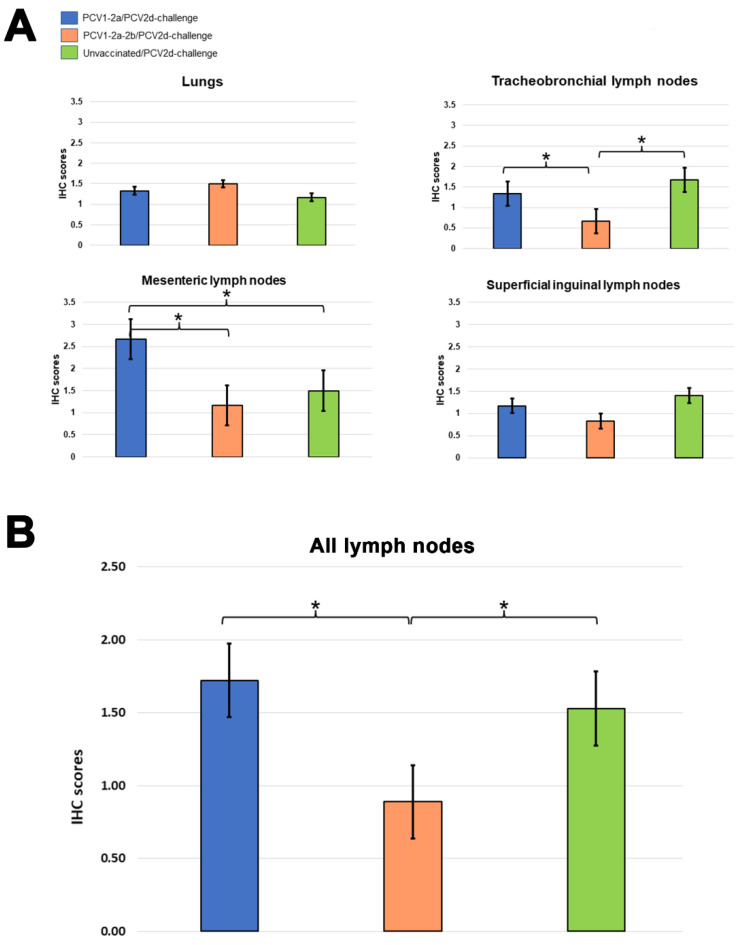
PCV2-positive immunohistochemical scoring of the lungs, tracheobronchial lymph nodes, mesenteric lymph nodes, and superficial inguinal lymph nodes of pigs. There were significant differences between the IHC scores of PCV1-2a-2b/PCV2d-challenge and unvaccinated/PCV2d-challenge pigs in the tracheobronchial lymph nodes (**A**). However, the average IHC immunolabelling-positive scores of all lymph nodes in each group were significantly different in comparisons made between the PCV1-2a-2b/PCV2d-challenge group and the PCV1-2a/PCV2d-challenge or unvaccinated/PCV2d-challenge groups (**B**). Data presented as values of mean ± standard error. Asterisks indicate statistically significant differences (* *p* < 0.05).

Microscopic findings indicated predominantly moderate to severe non-suppurative inflammation of the lungs, tracheobronchial lymph nodes, mesenteric lymph nodes, or superficial inguinal lymph nodes in the unvaccinated/PCV2d-challenge group when compared with the PCV1-2a or PCV1-2a-2b vaccinated groups (Supplementary Figure S1). Immunohistochemically, significant differences were observed between the immunolabelling-positive cells in the tracheobronchial lymph nodes and the mesenteric lymph nodes of the PCV1-2a-2b/PCV2d-challenge group and those of the PCV1-2a/PCV2d-challenge or unvaccinated/PCV2d-challenge groups (Figure 2 and Figure S2). Meanwhile, the IHC immunolabelling-positive score in the mesenteric lymph nodes of the PCV1-2a/PCV2d-challenge group was significantly higher than that of the other groups. Scores of the immunolabelling-positive cells in the lungs and superficial inguinal lymph nodes among the vaccinated and unvaccinated groups were not significantly different (Figure 2A). However, the average IHC immunolabelling-positive scores of all lymph nodes in each group were significantly different when comparisons were made between those of the PCV1-2a-2b/PCV2d-challenge group and those of the PCV1-2a/PCV2d-challenge or unvaccinated/PCV2d-challenge groups (Figure 2B).

### 3.2. Evaluation of PCV2d-Specific Neutralizing Antibody

**Figure 3:**
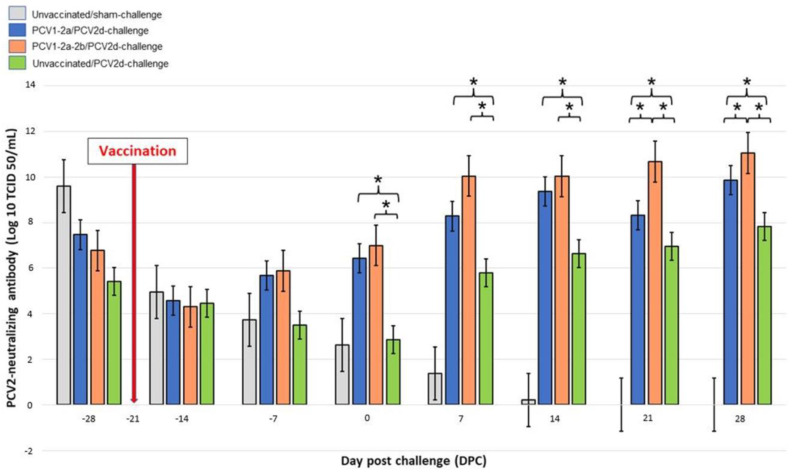
PCV2d-neutralizing antibody levels determined by immunoperoxidase monolayer assay (IPMA). Three weeks after vaccinations, neutralizing antibody levels of the vaccinated pigs increased and were found to be significantly different when compared with those of pigs of the unvaccinated/PCV2d-challenge group. Significant increases were observed in the PCV2-specific neutralizing antibodies of the PCV1-2a/PCV2d-challenge or the PCV1-2a-2b/PCV2d-challenge groups throughout the course of this study when compared with the unvaccinated/PCV2d-challenge group. Data are presented as mean ± standard error. Asterisks indicate statistically significant differences (* *p* < 0.05).

The immunoperoxidase monolayer assay (IPMA) for detection of the neutralizing antibody showed that a high amount of maternally derived antibodies (>log10^5^) was detected in pigs on the date of their arrival (Figure 3). This would indicate that these pigs were from non-clinically ill, PCV2-positive herds. Two weeks after administration of the PCV2 vaccine, the PCV2-specific neutralizing antibody levels of the vaccinated pigs increased and were found to be significantly different when compared with the positive control group on the day of the PCV2d challenge. Throughout the course of this study, significant changes in the PCV2-specific neutralizing antibody levels were observed in comparisons made between the PCV1-2a/PCV2d-challenge or the PCV1-2a-2b/PCV2d-challenge groups and the unvaccinated/PCV2d-challenge group (Figure 3). It should be noted that significant differences were observed in the neutralizing antibody levels in comparisons made between the PCV1-2a/PCV2d-challenge group and the PCV1-2a-2b/PCV2d-challenge group 3 weeks after the PCV2d challenge (6 weeks after vaccination). 

### 3.3. Evaluation of PCV2-Immunoglobulin G Antibody (IgG) Titers

**Figure 4:**
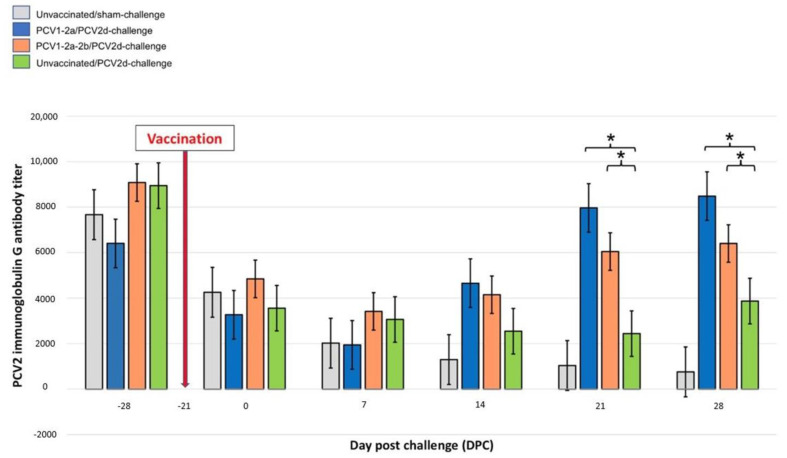
PCV2-immunoglobulin G (IgG) antibody titers of pigs when evaluated by ELISA. Serum samples were found to be positive for anti-PCV2 if the reciprocal ELISA titer was ≥1071. A significant upregulation of IgG titers in the PCV1-2a/PCV2d-challenge and PCV1-2a-2b/PCV2d-challenge pigs was observed at 21 DPC and onward when compared with the unvaccinated/PCV2d-challenge group. Data are presented as mean ± standard error values. Asterisks indicate statistically significant differences (* *p* < 0.05) when compared with the unvaccinated/PCV2d-challenge controls.

Serum samples were tested for PCV2-immunoglobulin G antibody (IgG) titers using the indirect ELISA technique. The results indicate that significant differences appeared at 21 days after inoculation, while the negative control group had a significantly lower amount of the PCV2-specific IgG antibody titers when compared with the PCV1-2a/PCV2d-challenge and the PCV1-2a-2b/PCV2d-challenge groups (Figure 4). The PCV1-2a/PCV2d-challenge and the PCV1-2a-2b/PCV2d-challenge groups had a similar level of PCV2-IgG antibody titers, while the PCV1-2a/PCV2d-challenge group had significantly higher PCV2-specific IgG antibody titers than did the positive control group. The IgG antibody titers at 28 days after inoculation revealed a trend similar to that observed at 21 days after inoculation, while the negative control also had a significantly lower amount of PCV2-IgG antibody titers when compared with the PCV1-2a/PCV2d-challenge and PCV1-2a-2b/PCV2d-challenge groups (Figure 4).

### 3.4. PCV2 DNA Detection in Serum Samples

The PCR results for determination of PCV2 viremia indicated that the unvaccinated/PCV2d-challenge pigs showed viremia on day 7 after inoculation, while pigs in the PCV1-2a/PCV2d-challenge and PCV1-2a-2b/PCV2d-challenge groups showed viremia on day 14 after viral inoculation. It should be noted that despite having viremic pigs in the PCV1-2a-2b/PCV2d-challenge groups, the number of PCV2-positive pigs in these groups was lower than that in the other PCV2d-challenge groups (Table 2).

**Figure 5:**
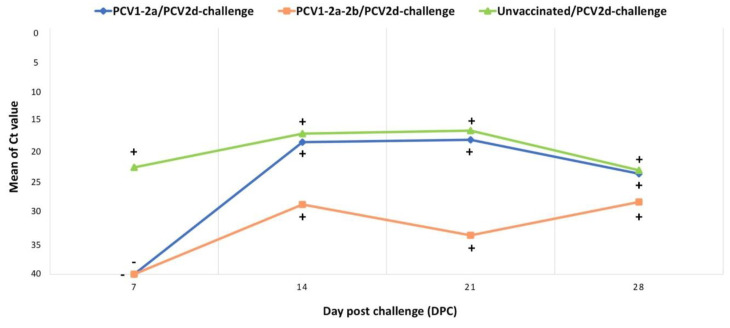
Determination of PCV2 viremia by real-time PCR. Mean Ct values indicated that pigs in the PCV1-2a-2b/PCV2d-challenge group had low levels of or no PCV2 viremia when compared with the other groups. At 7 DPC, no viremia was detected in pigs in either the PCV1-2a or the PCV1-2a-2b vaccinated groups.

Determination of mean PCV2 Ct values demonstrated that PCV1-2a-2b/PCV2d-challenge pigs exhibited low levels of PCV2 viremia when compared with the PCV1-2a/PCV2d-challenge and the unvaccinated/PCV2d-challenge groups (Figure 5). It should be noted that at 7 DPC, pigs in either the PCV1-2d vaccinated or the PCV1-2a-2b vaccinated groups showed no viremia when compared with the unvaccinated control pigs (Figure 5).

## 4. Discussion

In the present study, the chimeric PCV1-2a-2b-based vaccine demonstrated the best degree of efficacy in accelerating pig immune responses and reducing PCV2 viremia after being PCV2d-challenged. However, both the chimeric PCV1-2a-based vaccine and the PCV1-2a-2b-based vaccine managed to overcome the maternal derived antibody interference despite the presence of a high degree of maternal antibody prior to vaccination. Furthermore, the chimeric PCV1-2a and PCV1-2a-2b vaccines used in the present study could potentially reduce PCV2 viremia and, thereby, reduce the number of infected animals after being PDV2d-challenged when compared with the unvaccinated control group. However, the pigs vaccinated with the chimeric PCV1-2a-2b vaccine had significantly higher levels of PCV2d-specific neutralizing antibody titers when compared with pigs in the chimeric PCV1-2a vaccinated group. The in vivo study results corresponded to those of an in silico study that had employed T-cell epitope content comparison (EpiCC) analysis, which indicated the presence of the predicted T-cell epitopes among both the PCV2 commercial vaccine antigens and the heterologous genotypes of the PCV2 field strains [16]. Additionally, the PCV2a-2b combined antigens had a higher EpiCC score when compared with the PCV2a antigen type [16]. This could indicate that there is a possibility of greater enhancement of the immunological response by heterologous PCV2 antigens than by the homologous PCV2 antigen.

In agreement with previous reports [17,22,23,24,25], the experimental inoculation of PCV2d resulted in a mild degree of clinical signs. In fact, it has been well documented that economic losses due to PCV2 infection in pigs can mostly be associated with co-infection involving other pathogens [1,2]. As has been determined in this study, in spite of being housed in the ABSL-2 facility, microscopic findings in the lungs and lymph nodes of the PCV2d-inoculated and sham-inoculated pigs were found to exhibit mild to moderate degrees of inflammation. Importantly, the microscopic lesion scores could be used to evaluate the potency of the vaccines in this study, for which the IHC immunolabelling-positive score of all lymph nodes was lowest in the PCV1-2a-2b/PCV2d-challenge groups when compared with the others. On the other hand, immunohistochemistry, which is one of the most accurate standard diagnostic tests for PCV2 [26,27], should be used and applied for PCV2 viral antigen detection. However, it remains undetermined why significant differences in immunohistochemistry scores were observed in only the tracheobronchial lymph nodes and the mesenteric lymph nodes. Furthermore, the related organs, including the lungs and small intestines in both the vaccinated/PCV2d-challenge group and the unvaccinated/PCV2d-challenge groups, exhibited signs of inflammation in spite of the fact that no clinical signs were observed. Nevertheless, our study demonstrated that the PCV2a/2b antigen-based vaccinated group had the significantly lowest immunohistochemistry score when compared with the other challenged groups. Moreover, the PCV2a/2b antigen-based vaccinated group had the lowest number of infected samples in terms of superficial inguinal lymph nodes, tracheobronchial lymph nodes, and mesenteric lymph nodes.

Determination of growth performance among the studied pigs was not found to be significantly different. This could have resulted from the fact that the low virulence of the PCV2d strain that was used as an inoculum might not have produced obvious clinical signs and might not have strongly affect the health and performance of the pigs, especially over the short period of time in this study. However, the microscopic findings and the immunohistochemistry score indicated that members of all challenged groups were infected with PCV2d. In conclusion, the present study has demonstrated that both types of PCV2 vaccines, including the chimeric PCV1-2a-based vaccine and the chimeric PCV1-2a-2b-based vaccine, are effective against Thai PCV2d inoculation. 

## Figures and Tables

**Table 1:** Experimental design and vaccination plan in this study.

Group	Type of Vaccine	Dose
Unvaccinated/sham-challenge	Normal saline	2 mL, single dose
PCV1-2a/PCV2d-challenge	Chimeric PCV type 1-type 2a vaccine (Fostera^®^ PCV MetaStim, Zoetis, Charles city, IA, USA)	2 mL, single dose
PCV1-2a-2b/PCV2d-challenge	Chimeric PCV type 1, type 2a and type 2b vaccine (Fostera^®^ Gold PCV, Zoetis, Charles city, IA, USA)	2 mL, single dose
Unvaccinated/PCV2d-challenge	Normal saline	2 mL, single dose

**Table 2:** PCV2 DNA detection in the serum samples.

Group/ Challenge Day	Number of Positive Animals/Total Animals					
	−28	0	7	14	21	28
Unvaccinated/sham-challenge	0/6	0/6	0/6	0/6	0/6	0/6
PCV1-2a/PCV2d-challenge	0/6	0/6	0/6	4/6	4/6	3/6
PCV1-2a-2b/PCV2d-challenge	0/6	0/6	0/6	2/6	1/6	2/6
Unvaccinated/PCV2d-challenge	0/6	0/6	3/6	4/6	4/6	3/6

## Data Availability

Data is contained within the article and Supplementary Materials.

## Supplementary Materials

The following supporting information can be downloaded at: https://www.mdpi.com/article/10.3390/vetsci10020080/s1, Figure S1: Histopathological findings of the PCV1-2a and PCV1-2a-2b vaccinated groups when compared to the negative and unvaccinated/PCV2d-challenge groups. Figure S2: Immunohistochemical labelling of the PCV2 antigens in the lungs of negative control, PCV1-2a/PCV2d-challenge, PCV1-2a-2b/PCV2d-challenge and unvaccinated/PCV2d-challenge groups. Table S1: Average daily weight gain (ADG) of pigs.
