# Supplementary material for: Comparative Efficacy of Chimeric Porcine Circovirus (PCV) Vaccines against Experimental Heterologous PCV2d Challenges
Source: Vet Sci. 2023 Jan 21;10(2):80. doi: 10.3390/vetsci10020080 (PMC9959253; doi:10.3390/vetsci10020080)
Supplement: Supplementary file 1 [file vetsci-10-00080-s001.zip › vetsci-2115079-supplementary.pdf]

Supplementary materials

S1 Figure: Histopathological findings of the PCV1-2a and PCV1-2a-2b vaccinated groups when compared to the negative and unvaccinated/PCV2d-challenge groups. It is indicated that the moderate to severe non-suppurative interstitial pneumonia was observed in the unvaccinated/PCV2d-challenge group when compared to the negative control, PCV1-2a or PCV1-2a-2b vaccinated groups.

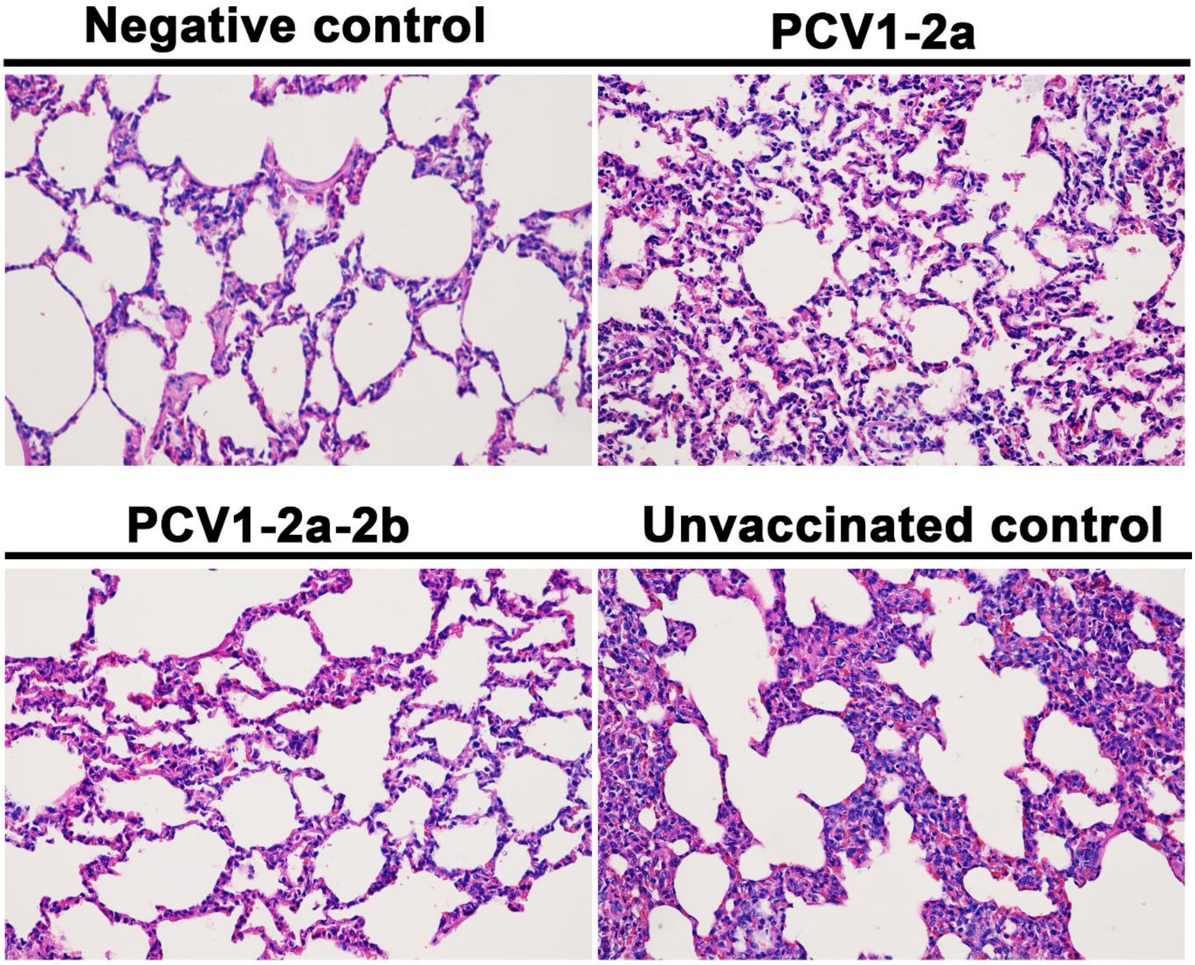

S2 Figure: Immunohistochemical labelling of the PCV2 antigens in the lungs of negative control, PCV1-2a/PCV2d-challenge, PCV1-2a-2b/PCV2d-challenge and unvaccinated/PCV2d-challenge groups. PCV2-antigen immunolabelling positive cells were observed in the macrophages of the unvaccinated/PCV2d-challenge positive control pig (inset).

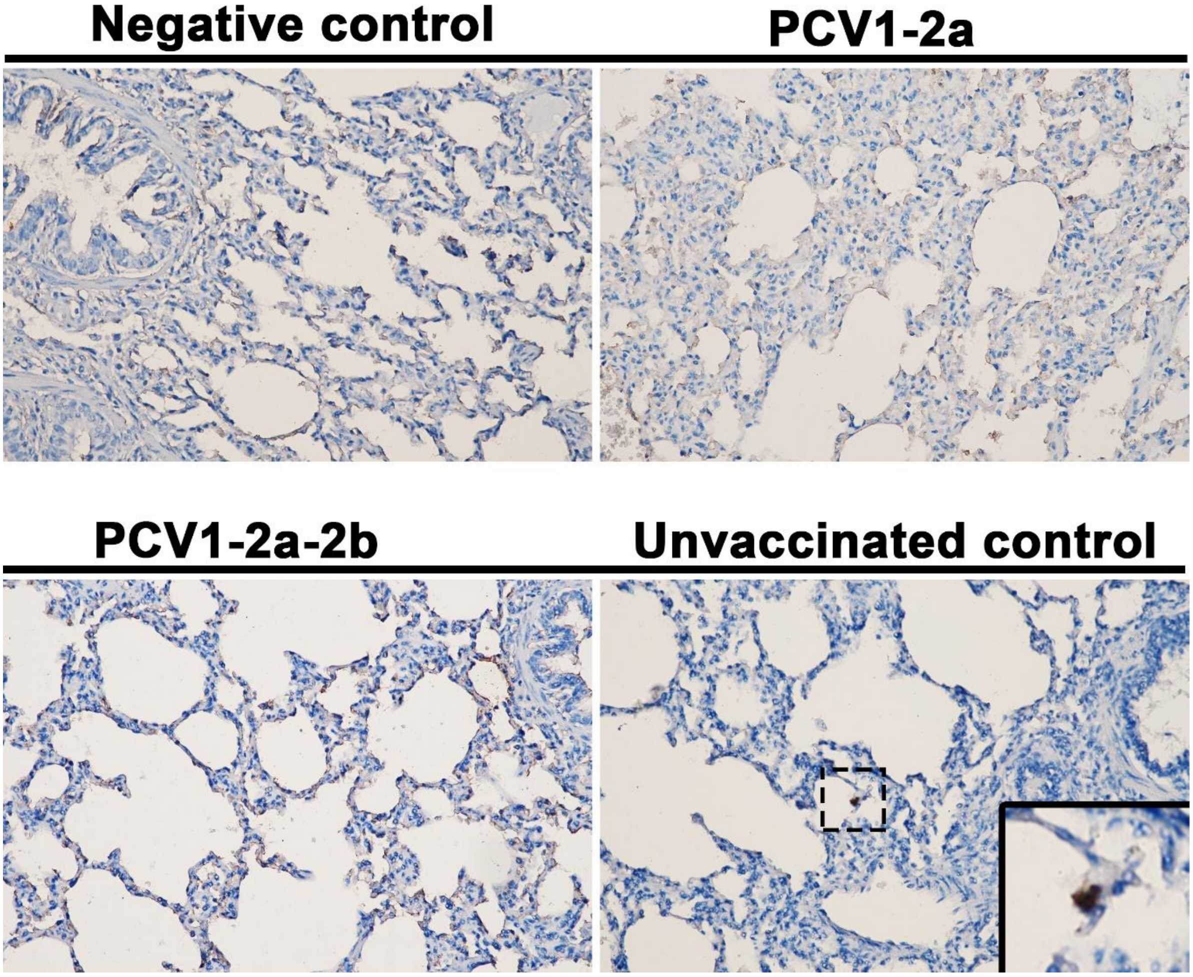

S1 Table: Average daily weight gain (ADG) of pigs. Vaccinated pigs with the chimeric PCV-vaccines tended to have a higher ADG than that of unvaccinated/sham-challenge or unvaccinated/PCV2d-challenge pigs, while the difference was not statistically significance.

Group	Mean (SE) of growth performance		
	Initial weight (kg)	Final weight(kg)	ADG (gram/day)
Unvaccinated/sham-challenge	8.16 (0.34)	39.75 (1.48)	560.94 (26.01)
PCV1-2a/PCV2d-challenge	7.03 (0.30)	39.58 (2.98)	577.98 (48.55)
PCV1-2a-2b/PCV2d-challenge	7.21 (0.18)	40.25 (2.42)	592.88 (43.47)
Unvaccinated/PCV2d-challenge	7.48 (0.48)	39.58 (2.25)	562.74 (33.46)

<u>Necropsy and sample collection of the animals</u>

Serum preparation

1. Blood was obtained from pigs in each group via sterile needle and syringe at 5 ml/pigs every week.
2. After sampling, the sterile syringes which contain whole blood were placed at the room temperature (25 ºC) to allow the separated serum from blood clot.
3. Collect serum from each syringe and placed in the centrifuge tube.
4. Centrifuge at 3,000 cycle/min. for 5 minutes.
5. Collect the serum from centrifuge tube, then kept sera in sterile tube and label individually.
6. Kept sera in -80ºC until the day of laboratory testing.

Pig necropsy procedures

1. After euthanized, examine the skin, eyes and ears of the carcass to detect the lesions.
2. Place the carcass on the table, examine the swelling of lymph node especially the superficial inguinal lymph nodes, then cut the skin from the abdomen side started from hindlimbs and open the abdomen by remove the skin of abdomen part.
3. Cut the ribs from the button part of ribs to the tops and carefully trim the diaphragm and muscle to expose the thoracic cavity.
4. Examine the internal organs and record abnormal lesions.
5. Cut the skin underneath the neck and chin then use scissor to cut through the mandibular symphysis and split the lower jaw, then detect for the abnormal lesions.
6. Grasp the tongue and pull it caudally. Then, cut the hyoid bones and dissect along the trachea and esophagus to detect for any abnormal secretion.
7. Examine inside the surface of trachea, esophagus to detect for any lesion which can be appeared.

8. Examine the tracheobronchial lymph nodes, lungs, pericardium and heart.
9. Open the trachea and main stem bronchi to examine the lesions.
10. Examine the pericardium and heart. Then, open the left and right ventricles and incise up into the atria and great vessels and examine the heart valves.
11. Grasp the mesenteric root and pull the viscera caudally, lifting the intestines and liver out of the abdomen and cutting the mesentery.
12. Examine the kidneys and the urogenital tract.
13. Dissect the liver from the intestines. Examine and bread slice the organ.
14. Cut the intestine at the mesenteric root. Then, cut both small and large intestine to detect for the abnormal lesions.
15. Cut the stomach and examine the surface inside.
16. Remove both kidneys from the abdominal cavity and cut in the half lengthways to examine the cortex, medulla and pelvis.
17. Examine the bladder. If there is evidence of urogenital disease, collect a sample of bladder into formalin and a urine sample into a sterile container.
18. Finally, Lung tissues, superficial inguinal lymph nodes, tracheobronchial lymph nodes and mesenteric lymph nodes were collected for microscopic evaluation and immunohistochemistry grading by cutting at the size 1x1 cm. and kept these tissues in 10% buffered formalin.

PCV2-immunoglobulin G antibody (Ig G) titers evaluation by Enzyme-linked immunosorbent assay (ELISA)

PCV2-immunoglobulin G antibody (Ig G) titer was done using the commercial ELISA test (BioChek®, USA).

Reagent preparation

1. **Substrate reagent:** To make substrate reagent, add 1 tablet to 5.5 ml of substrate buffer and allow to mix until fully dissolved (approximately 10 minutes). The prepared reagent should be made on day of use but will be stable for one week if kept in dark at +4 °C. Drop tablets into clean container and add appropriate volume of substrate buffer.
2. **Wash buffer:** Empty the contents of one wash buffer sachet into one liter of distilled or deionized water and allow to dissolve fully by mixing.
3. All other kit components are ready to use but allow them to come to room temperature (22-27 °C) before use.

Sample preparation (positive and negative kit controls do not require diluting)

Dilute each test sample 1:50 in sample diluent reagent.

1. Take 5 μl of sample and pipette into dilution plate recording the position of each sample on a template.
2. Add to these wells 245 μl of sample diluent reagent to make a 1:50 dilution.

Test procedure

1. Remove the coated plate from sealed bag and record location of samples on template.
2. Add 100 μl of negative control into wells A1 and B1.
3. Add 100 μl of positive control into wells C1 and D1.
4. Each sample is run in a single well. Add 100 μl of diluted 1:50 samples into the appropriate wells. Cover plate with lid and incubate at room temperature (22-27 °C) for 30 minutes.
5. Aspirate contents of wells and wash 4 times with wash buffer (350 μl per well). Invert plate and tap firmly on absorbent paper.

6. Add 100 µl of conjugate reagent into the appropriate wells. Cover plate with lid and incubate at room temperature (22-27 °C) for 30 minutes.
7. Repeat wash procedure as in 5.
8. Add 100 µl of prepared substrate reagent into the appropriate wells. Cover plate with lid and incubate at room temperature (22-27 °C) for 15 minutes.
9. Add 100 µl of stop solution to appropriate wells to stop reaction and read the assay within 30 minutes.
10. Blank the microtiter plate reader on air and record the absorbance of controls and samples by reading at 405 nm.

PCV2 DNA detection in serum samples by real time PCR

1. 150 µl of serum was transferred in the microcentrifuge tube.
2. 300 µl of lysis buffer was added.
3. The mixed solution was incubated at room temperature (15-25 ºC) for 10 minutes.
4. 300 µl of binding buffer was added and completely mix well by gently vortexing.
5. The spin column was placed in a provided 2 ml collection tube.
6. Lysates were loaded on the column and centrifuged at 13,000 rpm for 1 minute.
7. Solution in the collection tube was discarded. Then, the column was placed back in the same 2 ml collection tube.
8. 500 µl of washing buffer A was added to column and centrifuged for 1 minute at 13,000 rpm.
9. Solution in collection tube was discarded. Then, the spin column was placed back in the same 2 ml collection tube.
10. The tube was centrifuged for 1 minute at 13,000 rpm.
11. 60 µl of elution buffer was added directly onto the spin column membrane.
12. The mixed solution was incubated at room temperature for 1 minute.

13. The tube was centrifuged for 1 minute at 13,000 rpm.

14. 5 µl of eluted solution was used as template for PCR.

The process was performed using a thermocycler (Bio-Rad®). The program was done taking 5 µl of DNA as template and consisted on a reverse transcription of 2 min at 50 ºC, a degeneration phase at 95 ºC for 10 minutes and a total of 40 cycles, each cycle consisted of 25 second at 95 ºC for degeneration and 1 minute at 60 ºC for annealing/extension.
